# An Investigation of the Stillbirths at a Tertiary Hospital in Limpopo Province of South Africa

**DOI:** 10.5539/gjhs.v4n6p141

**Published:** 2012-09-24

**Authors:** Sam Thembelihle Ntuli, Ntambwe Malangu

**Affiliations:** 1 Department of Public Health Medicine, University of Limpopo, Polokwane Campus, South Africa; 2 Department of Epidemiology and Biostatistics, University of Limpopo, Medunsa Campus, South Africa

**Keywords:** stillbirth, prematurity births, South Africa

## Abstract

**Objective:**

To determine the stillbirth rate and identify the causal factors associated with it in a tertiary hospital.

**Methods:**

A retrospective review of records of women who had stillbirths at a tertiary hospital of the Limpopo Province was conducted. The study period was two years from January 1, 2009 to December 31, 2010. The hospital maternity registers were used to identify the women who gave birth during the study period. Data were collected using a data collection form designed for the study. The data collected included maternal age, parity, gestation, mode of delivery, obstetric complications, infant or foetal’s gender and weight; whether the birth was fresh stillbirth or macerated and cause of stillbirth.

**Results:**

There were 5597 deliveries during the two years period of the study. The hospital-based stillbirth rate was 38.4 per 1000 births, with 71% being macerated. The majority of women with stillborn infant in this study were in the age group (24%) 20-34 years, followed by (23%) aged 35 years and older. Nulliparity was associated with stillbirth. Unexplained intrauterine foetal death, hypertensive disease, placenta abruptio was the leading causes of stillbirth.

**Conclusion:**

In this study stillbirth rate seems to be unacceptably high, though less than those reported in other settings. The causal factors associated with it were identified as nulliparity, unexplained intrauterine foetal deaths, hypertensive disease, and placenta abruptio. Because of the high rate of stillbirths reported in this study, it is recommended that interventions be made to introduce fetal autopsies at the tertiary healthcare facilities and that an educational intervention aimed teaching pregnant women be instituted.

## 1. Introduction

Stillbirth continues to pose serious challenges in both developed and/or developing countries. In the recent years, a significant decline in stillbirths has occurred in many developed countries due to improvement in antenatal and delivery care, while rates in developing countries remain high. A number of studies have reported a stillbirth rate of less than 10 per 1000 delivery in developed countries (Shankar et al., 2002; O’Leary et al., 2007; McClure et al., 2007; Archibong et al., 2003), while other studies indicated rates of 10 to 40 per 1000 in developing countries (Shrestha & Yadav, 2010; Ngoc et al., 2006; Elhassan et al., 2009; Onyiriuka, 2009; Engmann et al., 2009; Chigbu et al., 2009; Saving Babies, 2009). However, there are few studies with a stillbirth rate of more than 40 per 1000 delivery in developing countries (Hossain et al., 2009; Jammeh et al., 2010; Onadeko & Lawoyin, 2003; Stanton et al., 2006).

There are different medical and nonmedical factors that result in pregnant women given birth to a stillborn baby. The most common medical factors reported are pre-eclampsia/eclampsia, obstetric haemorrhage and prolonged/obstructed labour (Elhassan et al., 2009; Engmann et al., 2009; Jammeh et al., 2010; McClure et al., 2009; Walch et al., 2008). A few studies reported placenta/cord factors, diabetes mellitus and congenital malformation as the commonest conditions (Shrestha & Yadav, 2010; Zupan, 2005). Maternal or foetal infection as possible cause of stillbirth has been reported in developing countries, whereas in developed countries lower proportion is attributable to infection (Gibb, 2002; Cham et al., 2009). Some studies reported advanced maternal age and multipara as high risk factors of stillbirth (O’Leary et al., 2007; Jammeh et al., 2010; Stanton et al., 2006). Hossain et al. (2009) in their study shows that gender of the baby was not associated with stillbirth.

Several studies reported nonmedical factors such as referral from peripheral health facility, delay in receiving appropriate management, lack of skilled birth attendants and antenatal care as the commonest conditions that lead to stillbirths (Shrestha & Yadav, 2010; Hossain et al., 2009; Onadeko & Lawoyin, 2003; Zupan, 2005). Jammeh and colleagues (2010) in their study reported caesarean section delivery as the risk factor of stillbirth, while other studies showed no relationship between the caesarean section delivery and stillbirth rate (WHO, 2004). Cham et al. (2009) from their study conducted in Gambian hospitals, reported vaginal delivery as a risk factor of stillbirth in women with severe obstetric complications.

The studies reviewed illustrated the variation in rates and the risk factors associated with stillbirths in developed and developing countries. Tertiary health centers usually admit more risk obstetric patients. There is a need to understand the distribution of fresh and macerated stillbirths and deaths within the immediate postpartum period in order to identify factors contributing to them and relevant interventions. This study was conducted to determine the rates and the documented causal factors of stillbirths among neonates at Pietersburg provincial referral hospital, South Africa.

## 2. Methods

A retrospective descriptive study was conducted at a provincial tertiary hospital in the Limpopo Province, South Africa. The study was performed over 2-years period (January 1, 2009 to December 31, 2010). The hospital maternity registers were used to identify the women who gave birth during the study period. Data were collected using a data collection form designed for the study. Births without information on vital status were excluded in this study. Stillbirth in this study was defined, in accordance with the World Health Organization’s International Classification of Disease (ICD-10) recommendation for international comparison, as the death of a foetus weighing at least 1000 g occurring after 28 weeks of gestation (WHO, 2004).

The Ethics Committee of the University of Limpopo (Medunsa Campus) approved the study; anonymity and confidentiality of patient personal information were protected through several mechanisms. Data for the study were collected using a pre-designed data collection form by trained nurse assistants. The data collected included maternal age, parity, gestation, mode of delivery, maternal obstetric complications, infant or foetal’s gender and weight, whether the birth was fresh stillbirth or macerated and cause of stillbirth. A stillbirth was defined as an intrauterine death of a fetus weighing at least 500 grams, whose gestation was over 20weeks, occurring before the complete expulsion or extraction from its mother. A fresh stillbirth was defined as the intrauterine death of a fetus during labor or delivery; while a macerated stillbirth was defined as an intrauterine death of a fetus occurring before the onset of labor and the fetus shows degenerative changes (WHO, 2001). The numbers of fresh and macerated stillbirths are presented as a proportion of all deliveries. Categorical data (i.e. referral status, mode of delivery, baby’s gender and weight) was displayed as percentages. Continuous data (i.e. maternal age and parity) was reported as mean ± SD. Comparison was performed using student t-test for continuous variables and chi-square test for categorical variables. The analysis was performed with Statistical software (STATA 9.0; StataCorp; College Station, TX). The p-values of less and/or equal to 0.05 were considered statistically significant.

## 3. Results

A total of 5597 deliveries were recorded during the period of the study (January 1, 2009 to December 31, 2010). Of these, 218 were stillbirths given a stillbirth rate of 38.9 per 1000 deliveries. The average age of the mother was 28.4 ± 6.9 years (range: 16 to 44 years). More than half (55%) of women were less than 30 years of age. Seventy four mothers (34%) were primigravida, 49(23%) primiparous, 66(31%) had parity between ranging from 2 to 4 children, 8(4%) had parity of 5 or more children, and the remaining 18(8%) parity was unspecified. Ninety-four women (43%) were referred from district, regional hospitals and private sector. The majority of these women were from district hospitals - 73/94(78%). Seventy five patients (34%) delivered by caesarean section and of these 46(61%) were macerated. The stillborn infants had the following characteristics. Fifty-one percent were female and 71% were macerated stillbirths. Their mean weight was 2178g ± 0.84 (Table 2). Of hundred and forty-one (65%) stillborn babies had a low birth weight (birth weight less than 2500g), the majority of them were macerated. There were some differences with regard to the profile of their mothers. The age of women who had fresh stillbirths was significantly lower than those of women who had macerated stillbirths (p<0.05). Additionally, the parity of the mothers with macerated stillbirths was significantly higher than those with fresh stillbirths (p<0.05). However, no significance difference was observed with regard to distance travelled to tertiary care services (p>0.05), although, of the 218 stillbirths, 94(43%) had been referred from other health care facilities. Although the difference was not statistically significant, referrals were slightly higher among women with fresh stillbirths than those with macerated stillbirths (Table 3).

**Table 1:** Maternal demographic characteristics

	N	%
Maternal age		
<20	20	9
20-24	51	24
25-29	47	22
30-34	36	17
35+	49	23
Unspecified	12	6
Parity		
0	74	34
1	49	23
2	30	14
3	24	11
4	12	6
5+	8	4
Unspecified	18	8

**Table 2:** Stillbirth characteristics

	N	%
Gender		
Male	108	49
Female	110	51
Birth weight		
1000-1449	61	28
1500-1999	39	18
2000-2499	41	19
≥2500	77	35

**Table 3:** Demographic characteristics between mothers with fresh and macerated stillborn babies

	Fresh Stillbirth	Macerated stillbirth	Test statistics	p-value
Maternal age, mean ±SD	25.9 ± 6.1	29.4 ± 6.9	-3.524*	<0.01
Parity, mean ±SD	1.03 ± 1.3	1.5 ± 1.6	-2.081*	0.04
Distance to facility, mean ±SD	84.7 ± 65.2 km	73.6 ± 66.3 km	1.132*	0.25
Referred				
Yes	32(50%)	62(40%)	1.749**	0.19
No	32(50%)	92(60%)		
Mode of delivery						
Caesarean section	29(45%)	46(30%)	4.778**	0.03
Normal vaginal delivery	35(55%)	108(70%)		
Baby’s gender						
Male	32(50%)	76(49%)	0.008**	0.93
Female	32(50%)	78(51%)		
Baby’s Weight						
<2500g	41(64%)	100(65%)	0.015**	0.90
≥2500g	23(36%)	54(35%)		

* Student t-test;

** Chi-square test

There were 154(71%) macerated stillbirths documented during the study period. Of these, half (50%) were unexplained intrauterine foetal deaths, 28(18%) were due to maternal hypertensive disease, and 21(14%) were as a result of placenta *abruptio*. Among the fresh stillbirths group, 20(32%) were due to maternal hypertensive disease, 15(24%) placenta *abruptio*, and 4(6%) were due to foetal distress (Table 4).

**Table 4:** Etiology of stillbirths

	Fresh Stillbirth	Macerated stillbirth	Total

	N(%)	N(%)	N(%)
**Maternal**			
Hypertensive disease	20(32%)	28(18%)	48(22%)
Diabetes mellitus	1(2%)	6(4%)	7(3%)
Cephalo-pelvic disproportion	2(3%)	2(1%)	4(2%)
Unspecified	3(5%)	5(3%)	8(4%)
**Foetal**			
Congenital anomalies	1(2%)	1(1%)	2(1%)
Foetal distress	4(6%)	0(0%)	4(2%)
Breaches	3(5%)	0(0%)	3(1%)
**Infection**	1(2%)	2(1%)	3(1%)
**Placenta**			
Placental *abruption*	15(24%)	21(14%)	36(17%)
Placenta *previa*	0(0%)	2(1%)	2(1%)
Cord accidents	3(5%)	10(6%)	13(6%)
**Unexplained IUFD**	11(17%)	77(50%)	88(40%)

## 4. Discussion

In our study, the stillbirth rate was 38.9 per 1000 delivery. The findings is similar to those studies which range between 30 and 40 per 1000 delivery (Shrestha et al., 2010; Ngoc et al., 2006; Elhassan et al., 2009; Onyiriuka, 2009; Engmann et al., 2009; Chigbu et al., 2009). Although in other studies the rates were reported to be less than 10 per 1000 delivery (Shankar et al., 2002; O’Leary et al., 2007; McClure et al., 2007; Archibong et al., 2003). The latter were exclusively conducted in developed countries where antenatal and obstetric care at peripheral referring hospitals is much improved compared to developing countries. In South Africa, few studies have estimated national and provincial stillbirth rates. The current perinatal care survey of South Africa (2006/07) recorded a national stillbirth rate of 24.4 per 1000 deliveires (Saving Babies, 2009). According to the District Health Barometer (2007/2008), the average stillbirth rate for South Africa was 23.0 per 1000 births, and two districts (i.e. rural and urban) of the Limpopo Province had a slightly higher rate than the national average (Day et al., 2009). It is important to note that the stillbirth rate reported in our study is higher than the national average. This finding suggests that the rate of stillbirths may be increasing in this rural province. However, the reported rate is less than the rates reported from other settings in developing countries (Bhattacharya et al., 2010; Ugboma & Onyearugha, 2012; Euzebus et al., 2011).

Previous studies reported that pre-eclampsia/eclampsia, obstetric haemorrhage, prolonged/obstructed labour were the most common risk factors for stillbirths (Shrestha et al., 2010; Elhassan et al., 2009; Onyiriuka, 2009; Engmann et al., 2009; Jammeh et al., 2010; Walch et al., 2008; Begum et al., 2010), while few studies indicated that placenta/cord factors, congenital malformation and infections were the most common cause of stillbirth (Ngoc et al., 2006; Jammeh et al., 2010; Zupan, 2005; Stanton et al., 2006). However, other studies have reported unexplained intrauterine foetal death as the commonest cause of stillbirths (Shankar et al., 2002; Shrestha et al., 2010; Zupan, 2005; Saving Babies, 2009; Day et al., 2009). The findings of our study confirm pre-eclampsia/eclampsia and placenta/cord factors as the common causes of stillbirth, although unexplained intrauterine foetal deaths were the most common.

In our study, younger mother’s age between 20 and 24 years and those women aged 35 years and older had high proportion of stillbirths. This finding is in contrast to studies conducted elsewhere (Katz et al., 2008), which found higher rates of stillbirth among adolescents. Others have found stillbirths in older women (O’Leary et al., 2007, Shrestha et al., 2010; Stanton et al., 2006), while some reported no difference in stillbirths rates by maternal age (Goldenberg et al., 2007).

Specifically, in our data women who had never had prior live births were at higher risk for stillbirths. Hossain and colleagues (2009) as well as Jammeh and co-workers (2010) indicated that both nulliparity and grand multiparity were significantly associated with stillbirths. A similar study reported higher parity as risk factor for stillbirths (Stanton et al., 2006). Several studies reported caesarean section as a risk factor for stillbirth (Onadeko & Lawoyin, 2003), while other studies reported no relationship between caesarean section and intrapartum stillbirth (Hossain et al., 2009; WHO, 2004). One study conducted in Gambian hospitals, reported vaginal delivery as a risk factor for stillbirths (Walch et al., 2008). In the present study, 55% of fresh stillbirths occurred among women who had normal vaginal delivery.

There are many other factors that contribute to higher stillbirth rates in a community. Previous studies have indicated that socioeconomic status and literacy also influence pregnancy outcomes (Bhattacharya et al., 2010; Engmann et al., 2009; Korde-Nayak & Gaikward, 2008). In our study, the socioeconomic status and educational level of the study participants were not assessed, however, a large number of these women came from rural villages with poor socioeconomic backgrounds.

In our study, the majority (71%) of the stillbirths were macerated. This finding is in contrast to several recent studies which found higher proportion of fresh stillbirth (McClure et al., 2007; Chigbu et al., 2009; Hossain et al., 2009; Stanton et al., 2006); while some older studies found similar results (Shankar et al., 2002). The average age of mothers with macerated stillbirths were significantly high than those mothers with fresh stillbirths. The high number of stillbirths in our study can be explained by the lack of proper antenatal care, late referrals, poor transport facilities, limited specialist obstetrician support, long distances to the referral hospital and inadequate emergency obstetric care at referring centers close to patient residences. To reduce this high macerated stillbirth rate, it will be necessary for the provincial health department to implement audit processes to identify areas for improvement in obstetric care at the district and regional referral hospitals.

This study suffered from some limitations including the fact that it was limited to two years only; a longer study period may have resulted in either a higher or lower stillbirth rates as calculated. Moreover, the study was conducted in a tertiary referral hospital which admits patients that could not be managed at lower level of care due to complications; therefore, the situation may not be probably a real reflection of the obstetric performance in the province as a whole. In addition, some of the patients admitted might have been in labour for a prolonged period of time and ran into difficulties at a peripheral facility well before being referred to the tertiary hospital, this could partly explain the occurrence of fresh stillbirths.

As with retrospective studies, any missing data from patient files affects the reliability of the data but this was minimized by reviewing the maternity delivery registers and the patient files over the study period. The history of antenatal care was not documented in most of the patient files; therefore, this was not included in the final analysis. The causes of stillbirth were based on clinical assessment of the attending medical doctor not on *post-mortem*. Fetal autopsy is the useful diagnostic procedure for information on the cause of deaths (Sliver et al., 2007; ACOG, 2009). More accurate data could have been available if fetal autopsies were conducted to determine the cause of deaths. However, the review assisted in addressing the research questions adequately and in providing data that can be used for further studies and in training healthcare providers.

## 5. Conclusion

In this study stillbirth rate seems to be unacceptably high, though less than those reported in other settings. The causal factors associated with it were identified as nulliparity, unexplained intrauterine foetal deaths, hypertensive disease, and placenta abruptio. Because of the high rate of stillbirths reported in this study, it is recommended that interventions be made to introduce fetal autopsies at the tertiary healthcare facilities and that an educational intervention aimed teaching pregnant women be instituted.
